# Linear endoscopic ultrasonography-guided biopsy forceps removal of a fishbone from the muscularis propria

**DOI:** 10.1055/a-2602-2738

**Published:** 2025-05-28

**Authors:** Lizhi Yi, Qin Wang, Yuqiang Xu, Jing Lu, Yuan Shen, Kaisheng Zhang, Zhengyu Cheng

**Affiliations:** 1 66561 Department of Gastroenterology, The Peopleʼs Hospital of Leshan, Leshan, China

A 76-year-old man was admitted to our hospital with 3 months of intermittent epigastric pain. An enhanced computed tomography scan revealed a 2-cm high density strip penetrating the gastric antrum. Esophagogastroduodenoscopy (EGD) found the mucosa of gastric antrum was normal. Linear endoscopic ultrasonography (EUS) showed a hyperechoic lesion with posterior shadowing in the muscularis propria ( Fig. 1 ).

**Fig. 1:**
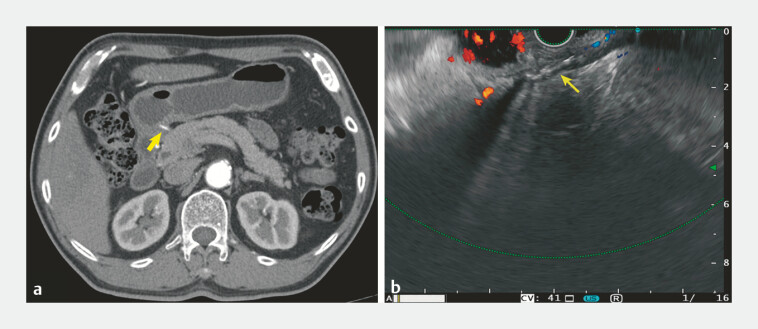
Appearance of the embedded fishbone on: **a** enhanced computed tomography scan, showing a high density strip penetrating the gastric antrum; **b** endoscopic ultrasonography, showing a hyperechoic lesion with posterior shadowing in the muscularis propria of the gastric antrum.

We firstly exposed the muscularis propria by endoscopic mucosal resection using band-ligation and then excised the submucosa of the specific area, which was marked by a snare under EUS guidance; however, the foreign body was still invisible. EUS was then performed again, and we observed one end of the foreign body was very close to the surface. EUS-guided biopsy forceps removal of the foreign body was performed ( Video 1 ). During the process, the foreign body was pushed out but, because no foreign body was seen on white-light imaging with the echoendoscope, an EGD was performed for further observation. The foreign body was found in the esophagus – we have surmised that it was pulled into the esophagus during withdrawal of echoendoscope ( Fig. 2 **a, b** ). Thereafter, EUS was performed again to observe whether there was any residual fish bone. To our surprise, we did find a residual hyperechoic strip in the muscularis propria, which was presumed to be another part of the fish bone ( Fig. 2 **c, d** ), and this part was also then removed. The patient had no discomfort postoperatively and was discharged 2 days after the procedure.

Linear endoscopic ultrasonography-guided biopsy forceps removal of a fishbone within the muscularis propria.Video 1

**Fig. 2:**
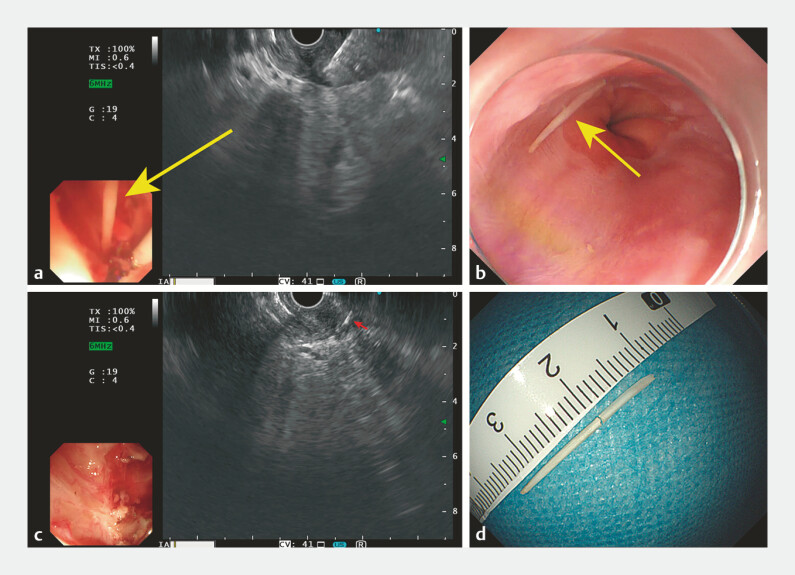
Images of endoscopic ultrasonography (EUS)-guided biopsy forceps removal of the two parts of a fish bone showing: **a** the first part of the fish bone pushed out when the biopsy forceps clamped the muscularis propria; **b** the first part of the fish bone, which was found in the esophagus on esophagogastroduodenoscopy; **c** a residual hyperechoic strip in the muscularis propria on repeat EUS; **d** the two parts of the fish bone after removal.

Successful removal of fish bones within the muscularis propria by full-thickness resection and endoscopic submucosal dissection have been reported 1 2 . Compared with these methods, the “cold removal” process used in our patient may minimize damage to the muscularis propria. For the first time, we show that EUS can be used to confirm complete removal of a fish bone.

Endoscopy_UCTN_Code_TTT_1AO_2AL

## Notes

Endoscopy E-Videos https://eref.thieme.de/e-videos
*E-Videos* is an open access online section of the journal *Endoscopy* , reporting on interesting cases and new techniques in gastroenterological endoscopy. All papers include a high-quality video and are published with a Creative Commons CC-BY license. Endoscopy E-Videos qualify for HINARI discounts and waivers and eligibility is automatically checked during the submission process. We grant 100% waivers to articles whose corresponding authors are based in Group A countries and 50% waivers to those who are based in Group B countries as classified by Research4Life (see: https://www.research4life.org/access/eligibility/ ). This section has its own submission website at https://mc.manuscriptcentral.com/e-videos .
